# Sap-Sucking Pests; They Do Matter

**DOI:** 10.3390/insects12040363

**Published:** 2021-04-19

**Authors:** David Wari, Kazumu Kuramitsu, Nickolas G. Kavallieratos

**Affiliations:** 1 Institute of Plant Science and Resources, Okayama University, Kurashiki, Okayama 710-0046, Japan; 2 Japan Society for the Promotion of Science, Chiyoda, Tokyo 102-0083, Japan; 3 Laboratory of Agricultural Zoology and Entomology, Department of Crop Science, Agricultural University of Athens, 75 Iera Odos str, Athens 11855, Attica, Greece

**Keywords:** Integrated Pest Management (IPM), management of sap-sucking pests, natural enemies, plant–insect interaction, plant–microbe–insect interactions, plant defense mechanisms, sap-sucking pest biology

This is an excerpt giving an overview of the Special Issue: Biology and Management of Sap-Sucking Pests. 

Generally, insect herbivores are grouped into categories based on their diet. Insect herbivores restricted to only one or a few closely related plant taxa, often of a single genus, are considered monophagous (or highly specialized) [1]. On the other hand, insect herbivores that feed on several plant species, usually within one botanical family, are referred to as oligophagous [1]. Furthermore, there are insect herbivores that feed on plant species of more than one plant family. These insect herbivores are known to be polyphagous (or highly generalized) herbivores. These groups of insects have been extensively studied and frequently reported to interact with plants in various ways [1]. Among these insect herbivores, there are the sap-sucking pests (comprising more than 75% of the pests known under monophagous, oligophagous, and polypagous herbivores [2]) that pose significant threats in commercial farms, either in open fields or closed systems.

Classic examples of hemipteran sap-sucking pests include aphids (Aphidoidea), lerps or psyllids (Psyllidae), scale insects (Coccidae), mealy bugs (Pseudococcidae), whiteflies (Aleyrodidae), leafhoppers (Cicadellidae), planthoppers (Delphacidae), cicadas (Cicadidae), stink or shield bugs (Pentatomidae), tarnished plant bugs (Miridae), squash bugs (Coreidae), and many more. A common feature among this diverse group of sap-sucking pests is the piercing–sucking mouthparts they use for feeding. Plant distortion, discoloration, or silvering of leaves in tomatoes by whiteflies (observations during studies in Wari et al. [3] and Saito et al. [4]), spotty yellow discolorations on the undersides of leaves, distorted, curled, or deformed leaves resulting in stunted growth, early leaf-fall, twig mortality on vegetables, shade trees and ornamental plantings caused by aphids, and hopperburn in rice by the brown planthopper (BPH) are a few of the examples triggered by the piercing–sucking mouthparts of the sap-sucking pests. In addition to weakening the plants through the sap they suck out, sap-sucking pests also act as vectors for a wide spectrum of viral diseases. Tomato yellow leaf curl virus (TYLCV), tomato chlorosis virus (ToCV), sweet potato chlorotic stunt virus, cowpea mild mottle virus (CpMMV), melon yellowing-associated virus (MYaV), lettuce infectious yellows virus (LIYV), tobacco mosaic virus, and tomato mosaic virus are but some of the viruses transmitted by the whiteflies [5]. Alfalfa mosaic virus, cucumber mosaic virus, potato virus Y, cauliflower mosaic virus, beet yellows virus, strawberry mottle virus, pea enation mosaic virus-1, barley yellow dwarf virus, potato leaf roll virus, carrot mottle virus, banana bunchy top virus, blueberry shoestring virus, and lettuce necrotic yellows virus are also economically significant viruses transmitted by aphids [6]. Similarly, rice tungro bacilliform, maize chlorotic dwarf, wheat dwarf, rice tungro spherical, rice transitory yellowing, maize sterile stunt, winter wheat mosaic, wheat rosette stunt, rice gall dwarf, rice wilted stunt, and rice black streaked dwarf are some of the viral diseases transmitted by leaf- and planthoppers [7]. Another common feature among sap-sucking pests, but lacking extensive research, is the excretion of waste product from their guts. Due to the ingestion of assimilate sugar-rich sap, large amounts of sticky sugar-rich residues, known as honeydew, are excreted. The excreted honeydew can sustain or contain various flora of microbes on leaf surfaces that can lead to a sooty appearance on the infected plants [8,9,10]. There is a vast spectrum of crop damage and viral, bacterial, and fungal diseases caused and vectored by the sap-sucking pests not listed herein. The case in point, a significant resemblance among these damages or the diseases caused and vectored by the respective sap-sucking pests, is the wide variety of economically important crops they infect. All these traits vary among sap-sucking pests, but the fact that their activity indirectly impacts food security, which can lead to a global food crisis, only ascertains their importance.

Sap-sucking pests have been progressing in their spread throughout the globe, affecting a vast array of crops at the same time as developing resistance against a wide-ranging spectrum of pesticides [11]. Therefore, innovative ideas for the development of alternative (pesticide-independent) methods to manage these sap-sucking pests are urgently needed. Globally, biological pest control methods have gained much attention due to their safety towards the environment, given that consumers have raised concerns related to synthetic chemicals. New tools are constantly being developed to assist the role of biological control agents in pest control. For example, geometric morphometrics and molecular analysis (COI barcode region) have recently shed light on the accurate identification of parasitoid species, especially those belonging to multiple complexes [12,13]. Such tools are of significant importance in optimizing the management of sap-sucking pests. However, pest control using biological control only is not feasible when pest outbreaks are severe and beyond control threshold limits. Integrated Pest Management (IPM), an ecosystem-based strategy that focuses on long-term maintenance of pests at economically accepted levels through the combination of techniques such as habitat manipulation, modification of cultural practices, use of resistant varieties, pesticides, and biological control agents, can improve pest control. Although pesticides may exhibit negative impacts to humans and the environment, they could play a major role in pest control, especially those that are harmful to pests but less-toxic to biological control agents (referred to as selective pesticides). Such pesticides are considered an integral arm of IPM and could play an important role in pest management. If the management of sap-sucking pests is to be successful, holistic and innovative approaches are needed, e.g., understanding the association among pesticides, sap-sucking pests, and their biological control agents. Selective pesticides in combination with biological control agents could be a good strategy for the successful management of sap-sucking pests. The recent rising trend of using plant-derived compounds to manage pests (e.g., BPH feeding and the release of honeydew from its gut, inducing direct and putative indirect defenses in rice, namely accumulation of phenolic compounds and release of volatile organic compounds that can serve to deter the BPHs and attract natural enemies, respectively [9,10]) could be an effective alternative against the sap-sucking pests. Moreover, studies on the behavior/ecology/taxonomy/life history/demography of the natural enemies of sap-sucking pests and extensive characterization of the viruses and pathogenic/symbiotic bacteria that are related to sap-sucking insects are also crucial. The deep knowledge of these approaches will build a strong scientific background for designing sustainable agricultural practices associated with the management of sap-sucking pests.

## Data Availability

Not applicable.
